# Supplementary material for: Fluorescent protein and peptide tags alter condensate formation and dynamics in vivo and in vitro
Source: EMBO Rep. 2025 Nov 20;27(1):89–121. doi: 10.1038/s44319-025-00626-y (PMC12796366; doi:10.1038/s44319-025-00626-y)
Supplement: Supplementary file 6 — Appendix [file 44319_2025_626_MOESM6_ESM.pdf]

Appendix

Fluorescent protein and peptide tags alter condensate formation and dynamics in vivo and in vitro

Kerstin Dörner, Michelle Jennifer Gut, Daan Overwijn, Fan Cao, Matej Siketanc, Stephanie Heinrich, Nicole Beuret, Justin Meyer, Timothy Sharpe, Kresten Lindorff-Larsen, Maria Hondele

Table of Contents

Appendix Figure S1	p.2
Appendix Figure S2	p.3
Appendix Figure S3	p.4
Appendix Figure S4	p.6
Appendix Figure S5	p.7
Appendix Figure S6	p.8
Appendix Figure S7	p.9
Appendix Figure S8	p.10
Appendix Figure S9	p.11
Appendix Figure S10	p.12
Appendix Figure S11	p.13
Appendix Figure S12	p.14

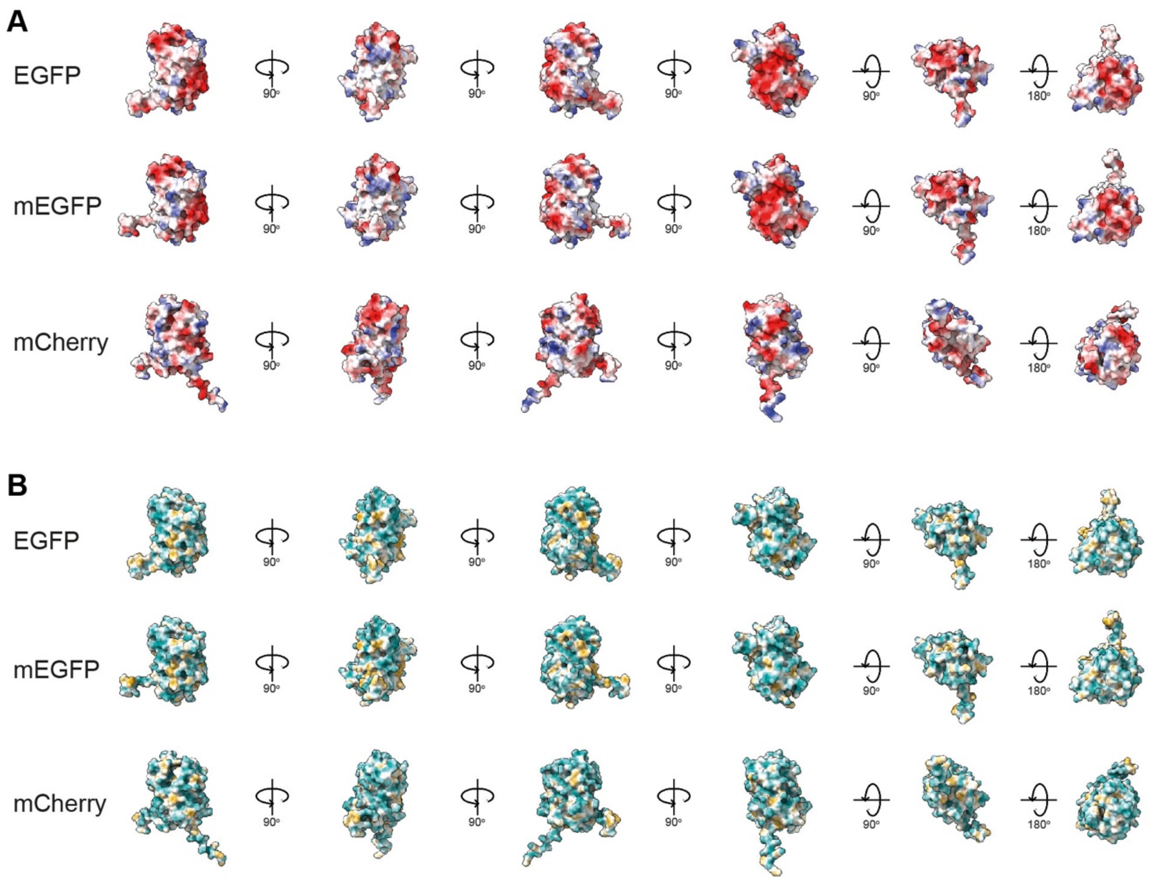

Appendix Figure S1:

(**A**) Surface charge of selected FPs from different angles, generated with AlphaFold v2.3. Positively charged patches are shown in red, negatively charged patches are shown in blue. (**B**) Surface Hydrophobicity of selected FPs from different angles, generated with AlphaFold v2.3. Hydrophobic patches are shown in beige; hydrophilic patches are shown in cyan.

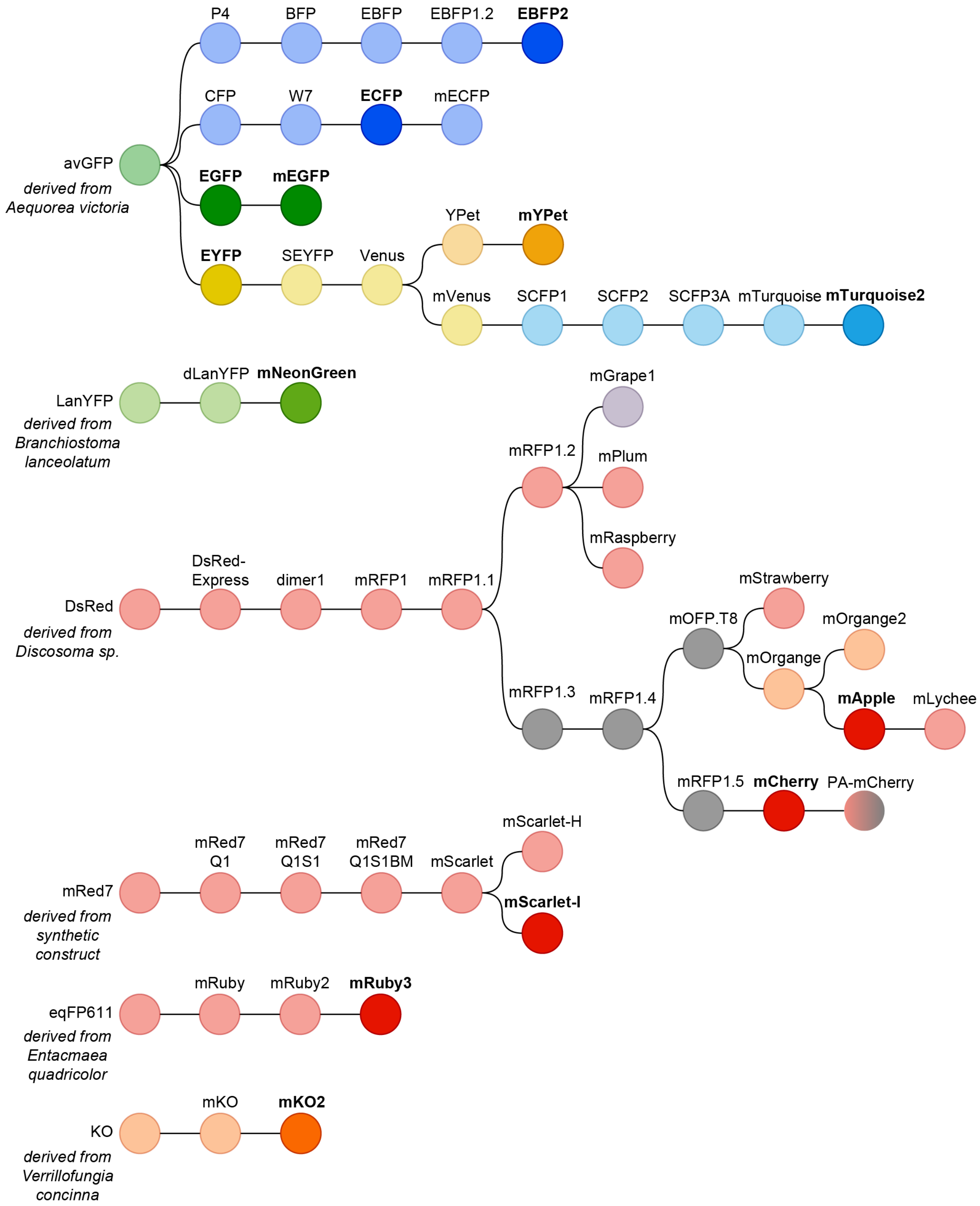

Appendix Figure S2:

Overview of the evolutionary relationship among selected fluorescent proteins based on FPbase.org. FP tags used in this study are displayed in bold.

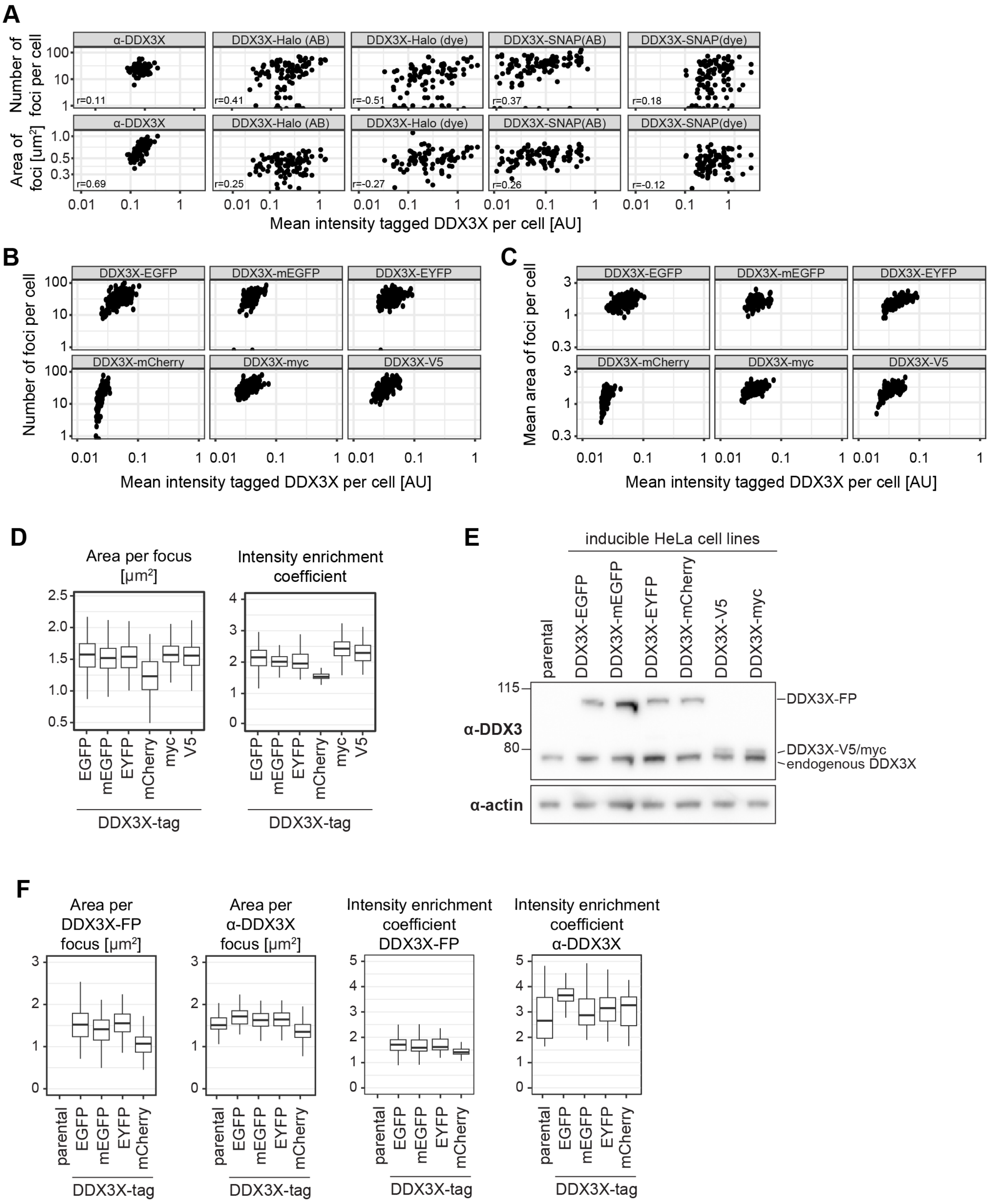

Appendix Figure S3:

(**A**) HeLa K cells were transiently transfected with DDX3X-Halo/SNAP plasmids for 24 h, and stressed with 500 µM sodium arsenite for 30 min before PFA fixation. Halo and SNAP tags were either visualized by incubation with respective dyes (dye) or by immunostaining (AB). Quantification of DDX3X foci as displayed in Figure 1C: number and area relative to DDX3X-Halo/SNAP expression level. Pearson correlation coefficient (r) is indicated. N = 3, n ≥ 90 cells.

(**B/C**) Stable inducible HeLa cell lines were induced with doxycycline for 24 h to express the respective DDX3X-tag constructs at endogenous level. Cells were stressed with 500 µM sodium arsenite for 30 min before PFA fixation. Quantification of DDX3X foci as displayed in Figure 1E: number (B) and area (C) relative to DDX3X-tag expression level. N = 3, n ≥ 105 cells.

(**D**) DDX3X-tag expression levels of stable inducible HeLa cell lines in Figure 1E, Quantification N = 3, n ≥ 105 cells.

(**E**) DDX3X-tag expression levels of stable inducible DDX3X-tag HeLa cell lines were analyzed by immunoblotting with the indicated antibodies.

(**F**) Stable inducible HeLa cell lines were induced with doxycycline for 24 h to express the respective DDX3X-tag constructs. Cells were stressed with 500 µM sodium arsenite for 30 min. Cells were also stained for DDX3X with immunostaining. Additional quantifications for images displayed in Fig. EV1E. N=3. n≥ 40 cells.

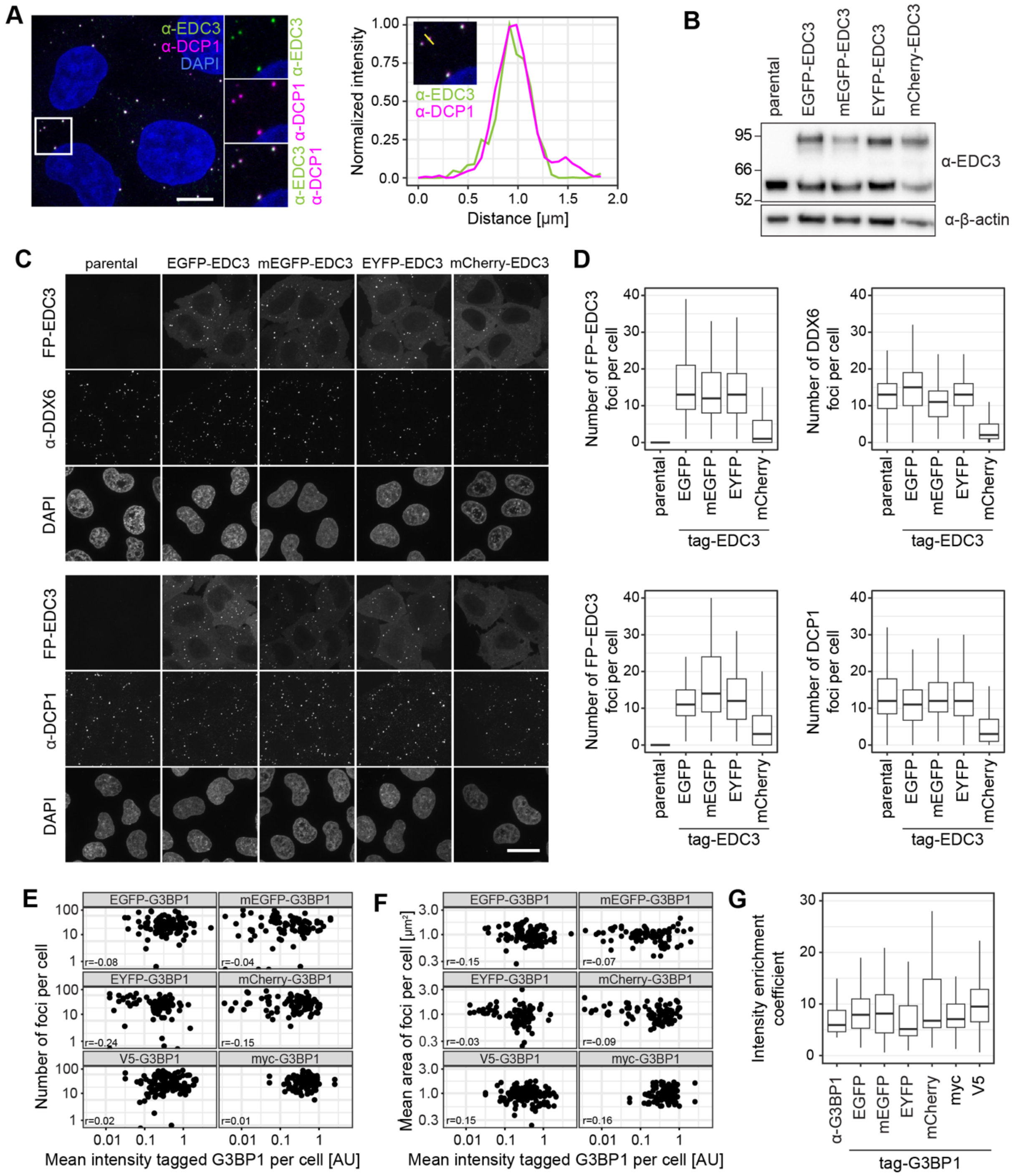

Appendix Figure S4:

(**A**) Stressed HeLa K cells (500 µM sodium arsenite, 30 min) were fixed with methanol and immunostained for EDC3 and DCP1 with respective antibodies. Scale bar: 10 µm. Line plot shows the normalized intensity of EDC3 and DCP1 across a P-body.

(**B**) Tag-EDC3 expression levels of stable inducible HeLa cell lines from Figure 3C were analyzed by immunoblotting with the indicated antibodies.

(**C/D**) Stable inducible HeLa cell lines were induced with doxycycline for 24 h to express the respective EDC3-tag constructs. Cells were stressed with 500 µM sodium arsenite for 30 min

before fixation. Cells from the same well were either immunostained for DDX6 or DCP1 with respective antibodies. Scale bar: 20 µm. Quantification of number of DDX6 and DCP1 foci (I). N = 3, n ≥ 140 cells.

(E/F/G) HeLa K cells were transiently transfected with G3BP1 plasmids for 24 h and stressed with 500 µM sodium arsenite for 30 min. V5-/myc-G3BP1 were visualized by immunostaining with the respective antibodies. Untransfected cells were immunostained for G3BP1 as a control. Quantification of G3BP1 foci as displayed in Figure 3E: Number (E) and area (F) relative to the cellular expression level Pearson correlation coefficient (r) is indicated. (G) Intensity enrichment coefficient (median intensity per focus/ median intensity of the cell). N = 3, n ≥ 90 cells.

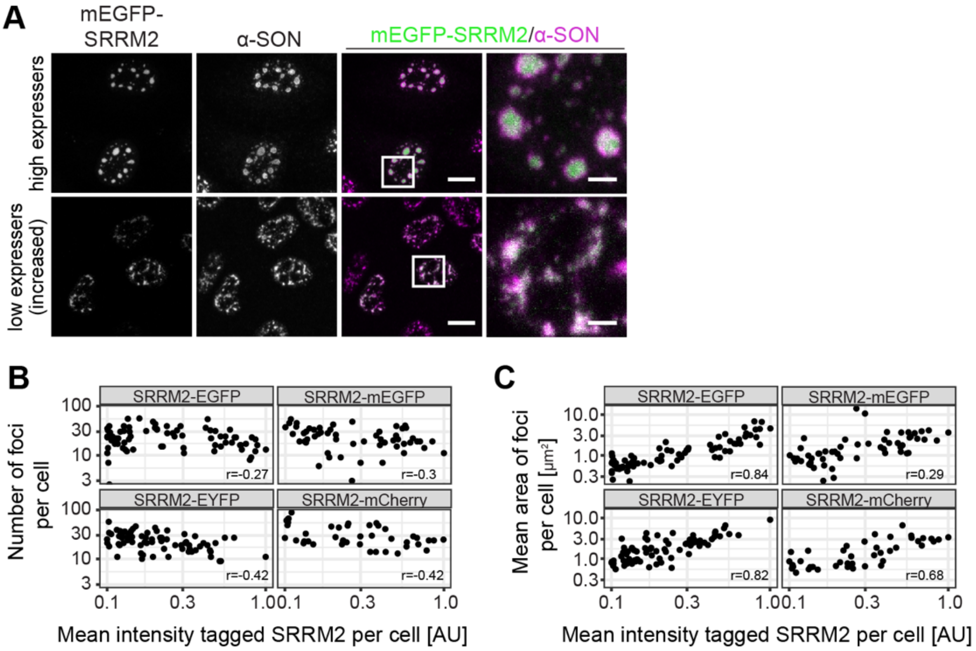

Appendix Figure S5:

(**A**) HeLa K cells were transiently transfected with a plasmid for expression of mEGFP-SRRM2. After 24 h, cells were stressed with 500 µM sodium arsenite for 30 min. For visualization of nuclear speckles cells were additionally immunostained for SON. Representative central plane images are shown. Scale bar: 10 µm, for Zoom-in 2 µm.

(**B/C**) HeLa K cells were transiently transfected with SRRM2 plasmids for 24 h. Quantification of SRRM2 foci of cells in Figure 3G: number (C) and area (D) relative to the cellular expression level. Pearson correlation coefficient (r) is indicated. N = 3, n ≥ 40.

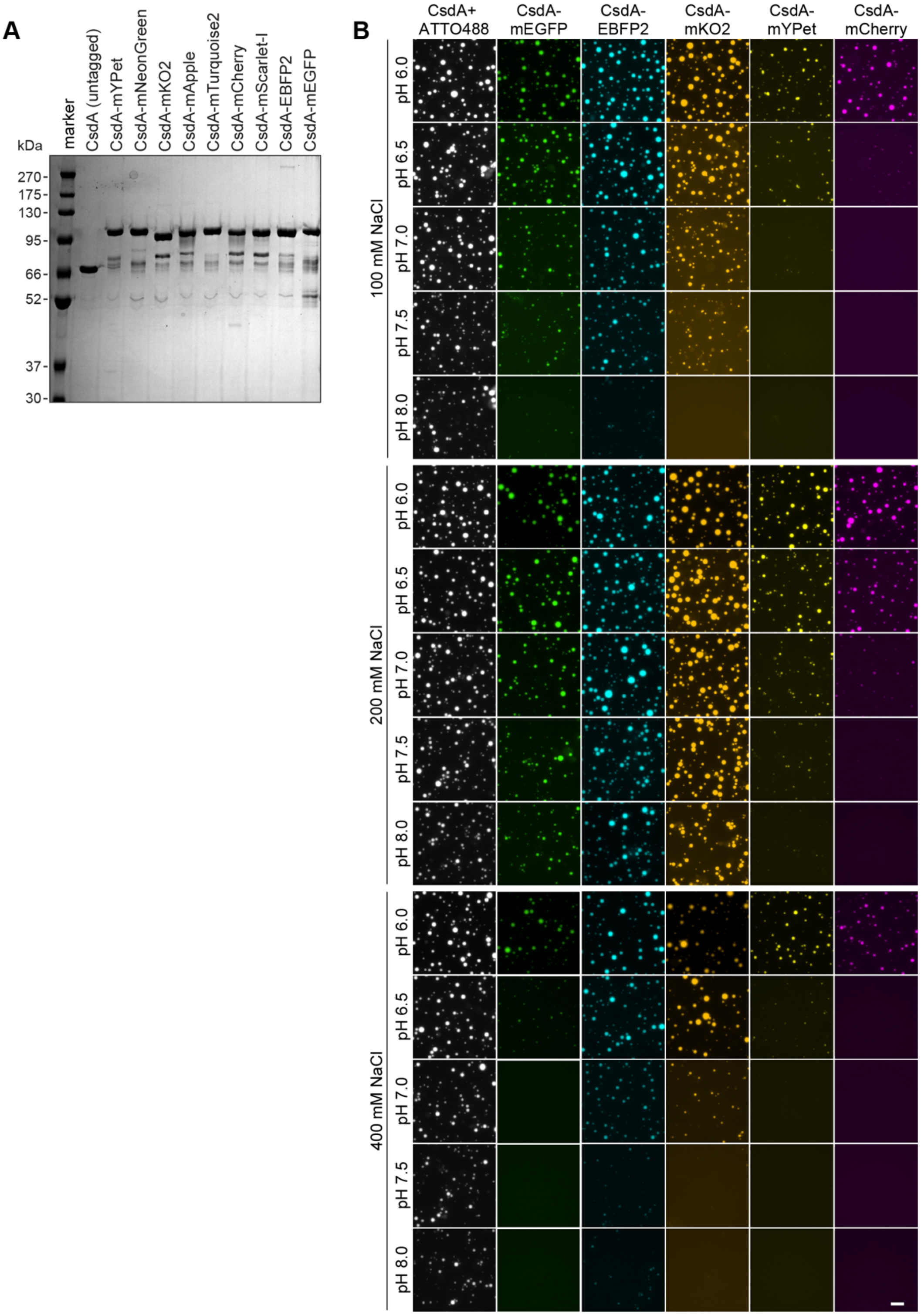

Appendix Figure S6

(**A**) Coomassie stained SDS PAGE with 0.5 µg of indicated CsdA protein per lane.

(**B**) *In vitro* condensation assay with 2 µM CsdA (tagged or untagged + 1% ATTO488-CsdA spike-in) in 25 mM sodium phosphate buffer at the indicated pHs and NaCl concentrations, 2 mM $MgCl_2$, 0.5 mg/mL BSA, 0.05 mg/mL poly(U). Incubated at 25°C for 1 h before imaging. Representative images are shown. Scale bar: 20 µm.

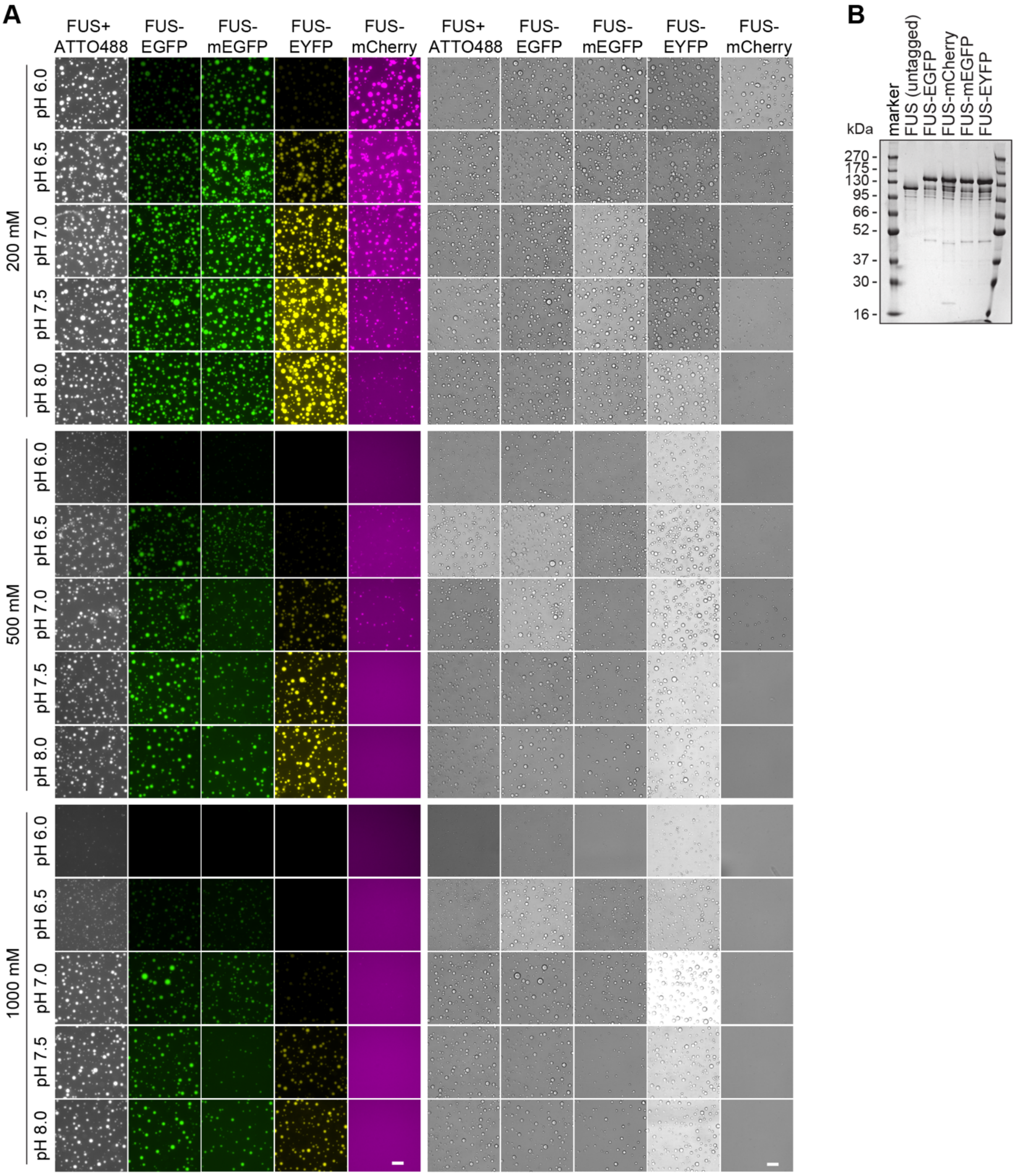

Appendix Figure S7

(**A**) *In vitro* condensation assay with 8 µM FUS (tagged or untagged + 2% ATTO488-FUS spike-in) in 25 mM sodium phosphate buffer at the indicated pHs and NaCl concentrations, 2 mM $MgCl_2$. Condensation was induced by addition of 10 µM His-3C protease removing the solubility tag. Incubated at 25°C for 30 min before imaging. Representative images are shown. Scale bar: 20 µm.

(**B**) Coomassie stained SDS PAGE with 0.5 µg of indicated FUS protein per lane.

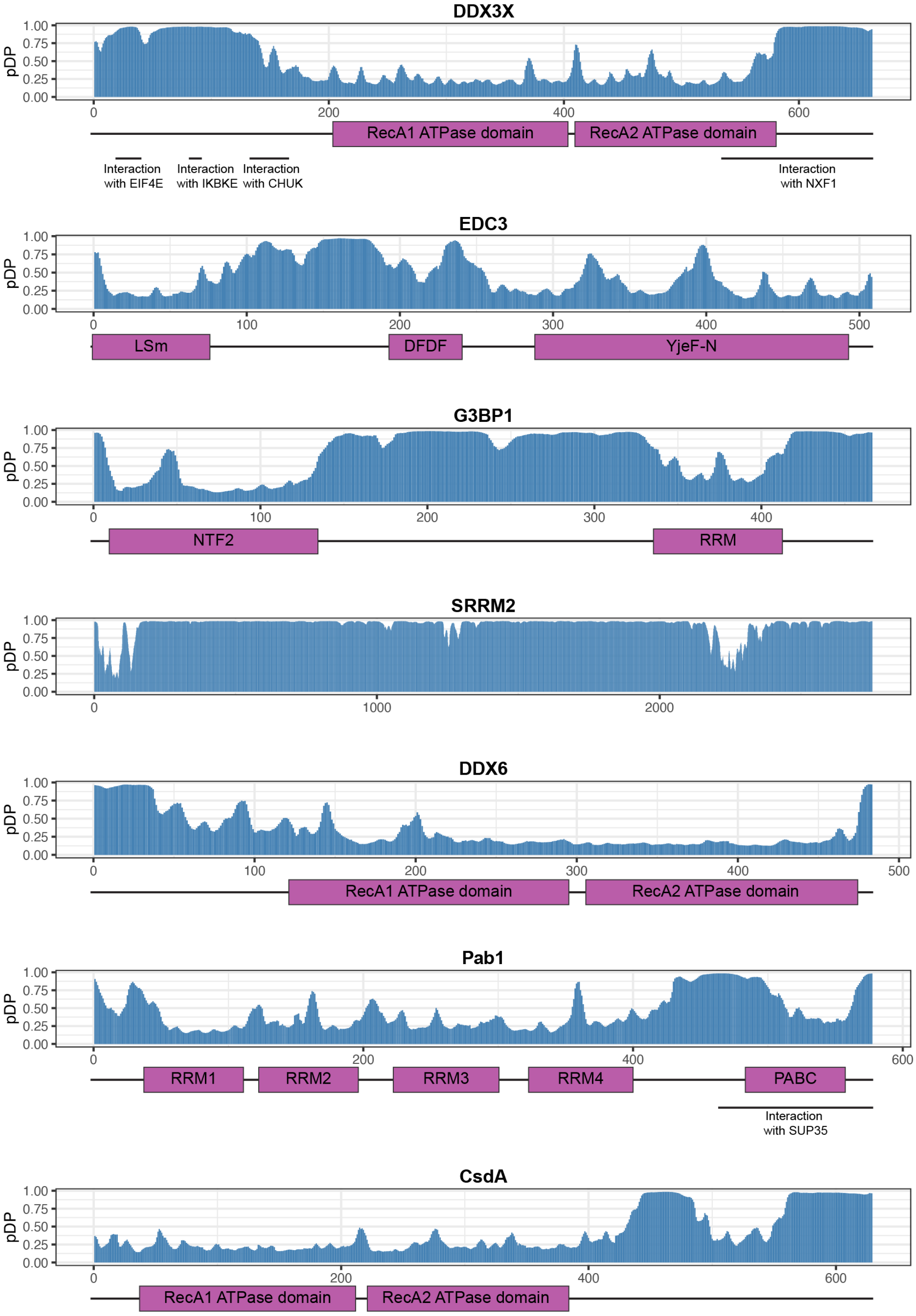

Appendix Figure S8

Predicted intrinsically disordered regions (based on FuzDrop) and functional domains (based on UniProt) of proteins used in this study.

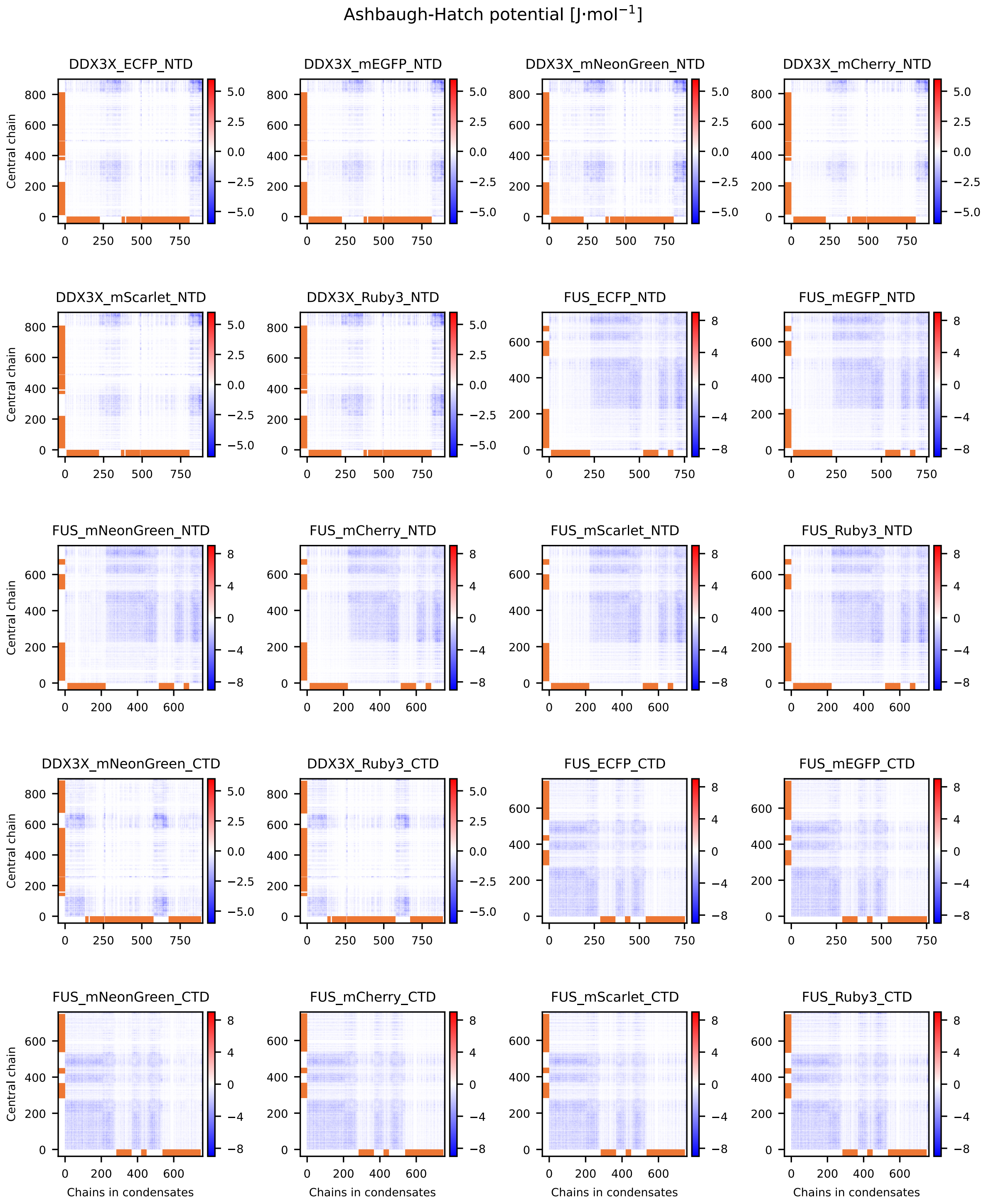

Appendix Figure S9

Averaged residue-pair interaction energies (the Ashbaugh-Hatch potential in forcefield) between the most central chain and the rest of the condensate for DDX3X and FUS with six different fluorescent proteins. The orange lines indicate folded parts. We only show the energy maps of constructs that form stable condensates during simulations.

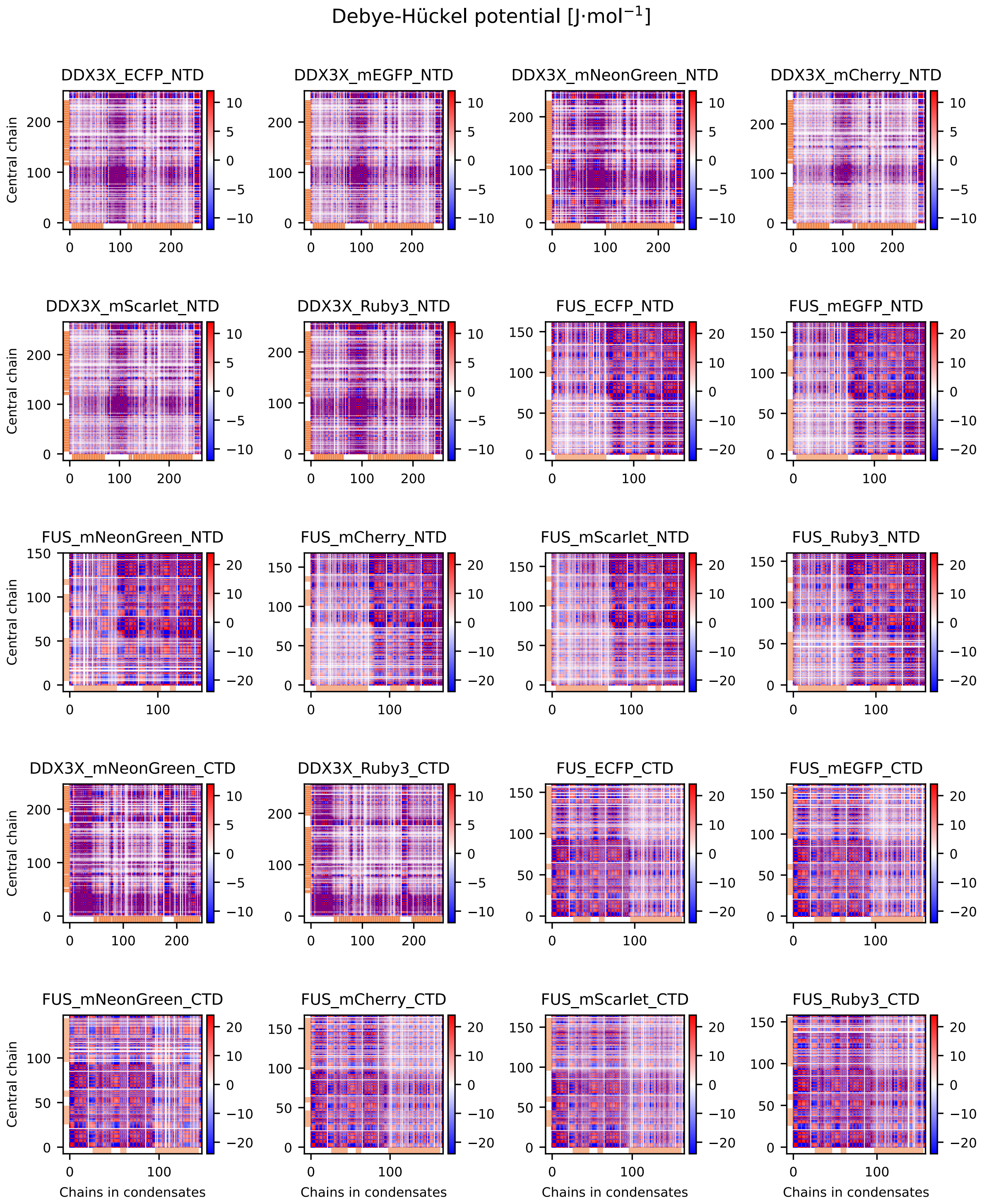

Appendix Figure S10

Averaged residue-pair electrostatic interaction energies (the Debye-Hückelpotential in forcefield) between the most central chain and the rest of the condensate for DDX3X and FUS with six different fluorescent proteins. Only the charged residues are shown. The orange lines indicate folded parts. We only show the energy maps of constructs that form stable condensates during simulations.

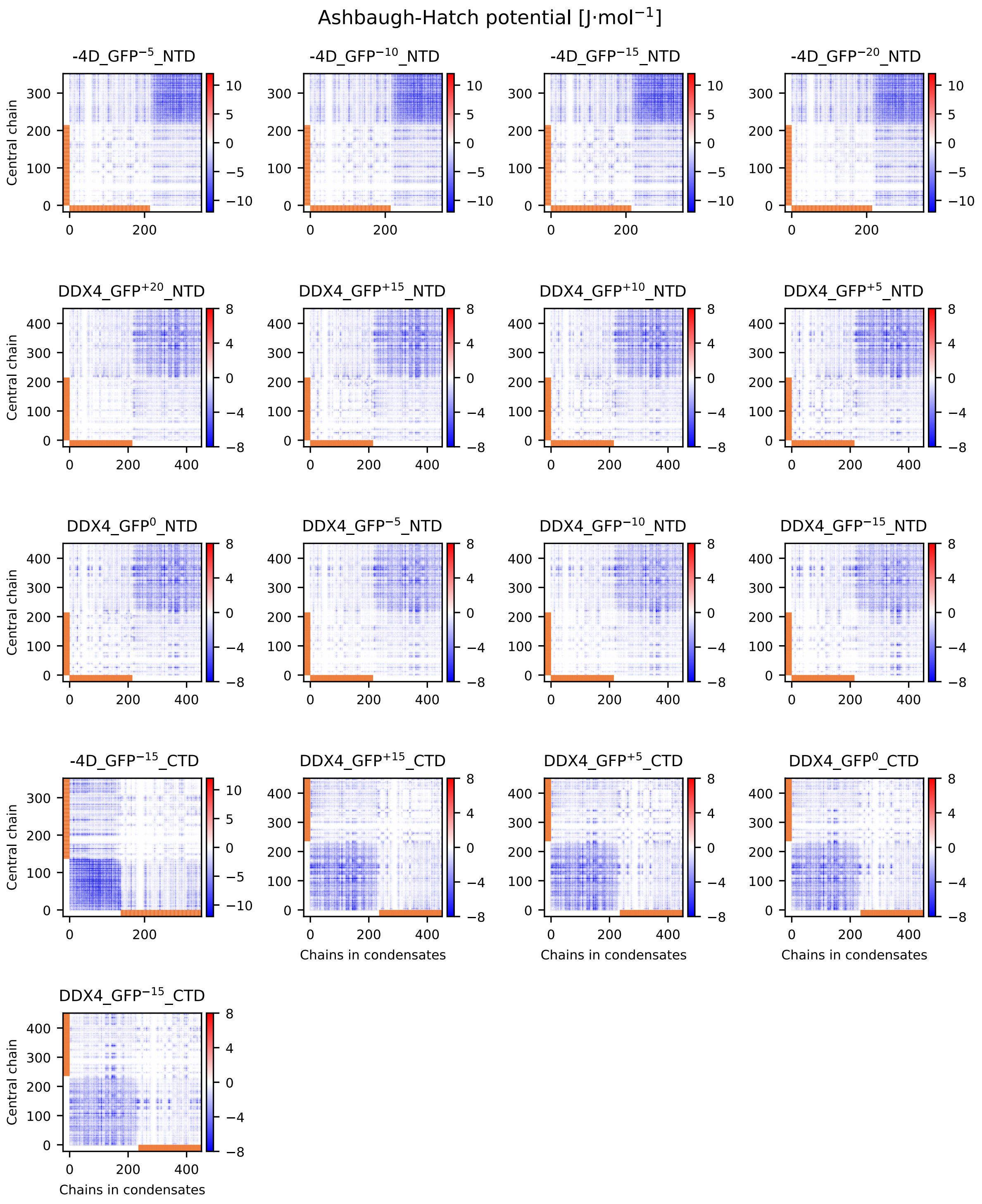

Appendix Figure S11

Averaged residue-pair interaction energies (the Ashbaugh-Hatch potential in forcefield) between the most central chain and the rest of the condensate for -4D and DDX4 with different GFP variants. The orange lines indicate folded parts.

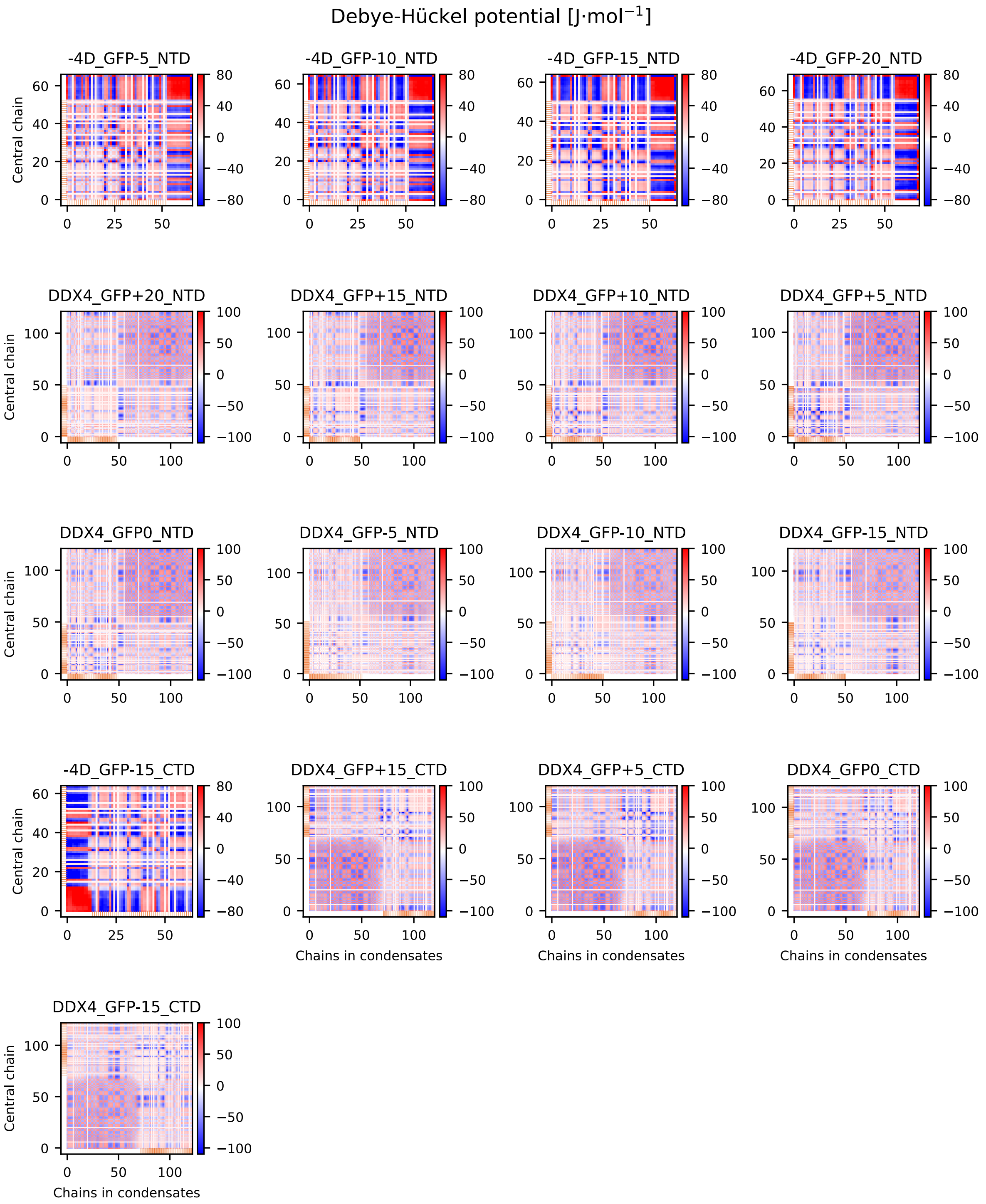

Appendix Figure S12

Averaged residue-pair electrostatic interaction energies (the Debye-Hückel potential in forcefield) between the most central chain and the rest of the condensate for -4D and DDX4 with different GFP variants. Only the charged residues are shown. The orange lines indicate folded parts. We only show the energy maps of constructs that form stable condensates during simulations.
