# Supplementary material for: Fluorescent protein and peptide tags alter condensate formation and dynamics in vivo and in vitro
Source: EMBO Rep. 2025 Nov 20;27(1):89–121. doi: 10.1038/s44319-025-00626-y (PMC12796366; doi:10.1038/s44319-025-00626-y)
Supplement: Supplementary file 15 — Expanded View Figures [file 44319_2025_626_MOESM15_ESM.pdf]

Expanded View Figures

Figure EV1. DDX3X tag effects are not correlated with expression level and evident in live cells.

(**A**, **B**) HeLa K cells were transiently transfected with DDX3X plasmids for 24 h, and stressed with 500 µM sodium arsenite for 30 min before fixation. Quantification of DDX3X foci as displayed in Fig. 1A: number (D) and area (E) relative to DDX3X-tag expression level. Pearson correlation coefficient (r) is indicated. $N = 3$, $n \geq 135$ cells. (**C**, **D**) Stable inducible HeLa cell lines were induced with doxycycline for 24 h to express the respective DDX3X-tag constructs. Cells were stressed with 500 µM sodium arsenite for 30 min and imaged live. Scale bar: 20 µm. Quantification of the number of DDX3X foci and their area (**D**). Box plots show the median (center line), 25th–75th percentiles (bounds of box), and whiskers=1.5x IQR. $N = 3$, $n \geq 110$ cells. (**E**, **F**) Stable inducible HeLa cell lines were induced with doxycycline for 24 h to express the respective DDX3X-tag constructs. Cells were stressed with 500 µM sodium arsenite for 30 min. Cells were also stained for DDX3X with immunostaining. Scale bar: 20 µm. Quantification of the number of FP-DDX3X and DDX3X foci detected by the DDX3X antibody (**F**). Box plots show the median (center line), 25th–75th percentiles (bounds of box), and whiskers=1.5x IQR. $N = 3$. $n \geq 40$ cells.

© The Author(s)

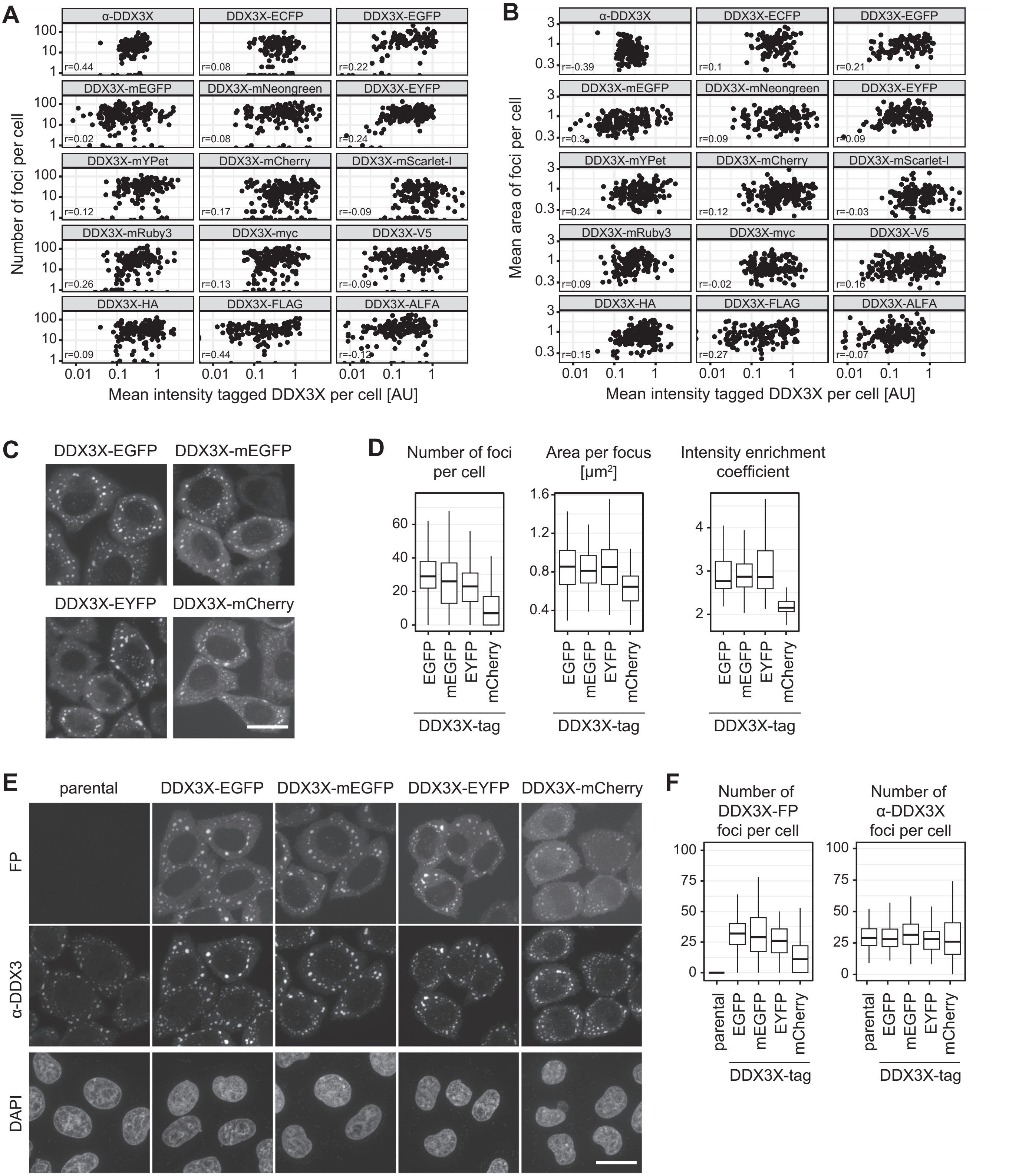
A
B
α-DDX3X
DDX3X-ECFP
DDX3X-EGFP
DDX3X-mEGFP
DDX3X-mNeongreen
DDX3X-EYFP
DDX3X-mYPet
DDX3X-mCherry
DDX3X-mScarlet-I
DDX3X-mRuby3
DDX3X-myc
DDX3X-V5
DDX3X-HA
DDX3X-FLAG
DDX3X-ALFA
Number of foci per cell
Mean area of foci per cell
Mean intensity tagged DDX3X per cell [AU]
C
DDX3X-EGFP
DDX3X-mEGFP
DDX3X-EYFP
DDX3X-mCherry
D
Number of foci per cell
Area per focus [μm²]
Intensity enrichment coefficient
EGFP
mEGFP
EYFP
mCherry
DDX3X-tag
E
parental
FP
α-DDX3
DAPI
F
Number of DDX3X-FP foci per cell
Number of α-DDX3X foci per cell

 © The Author(s)

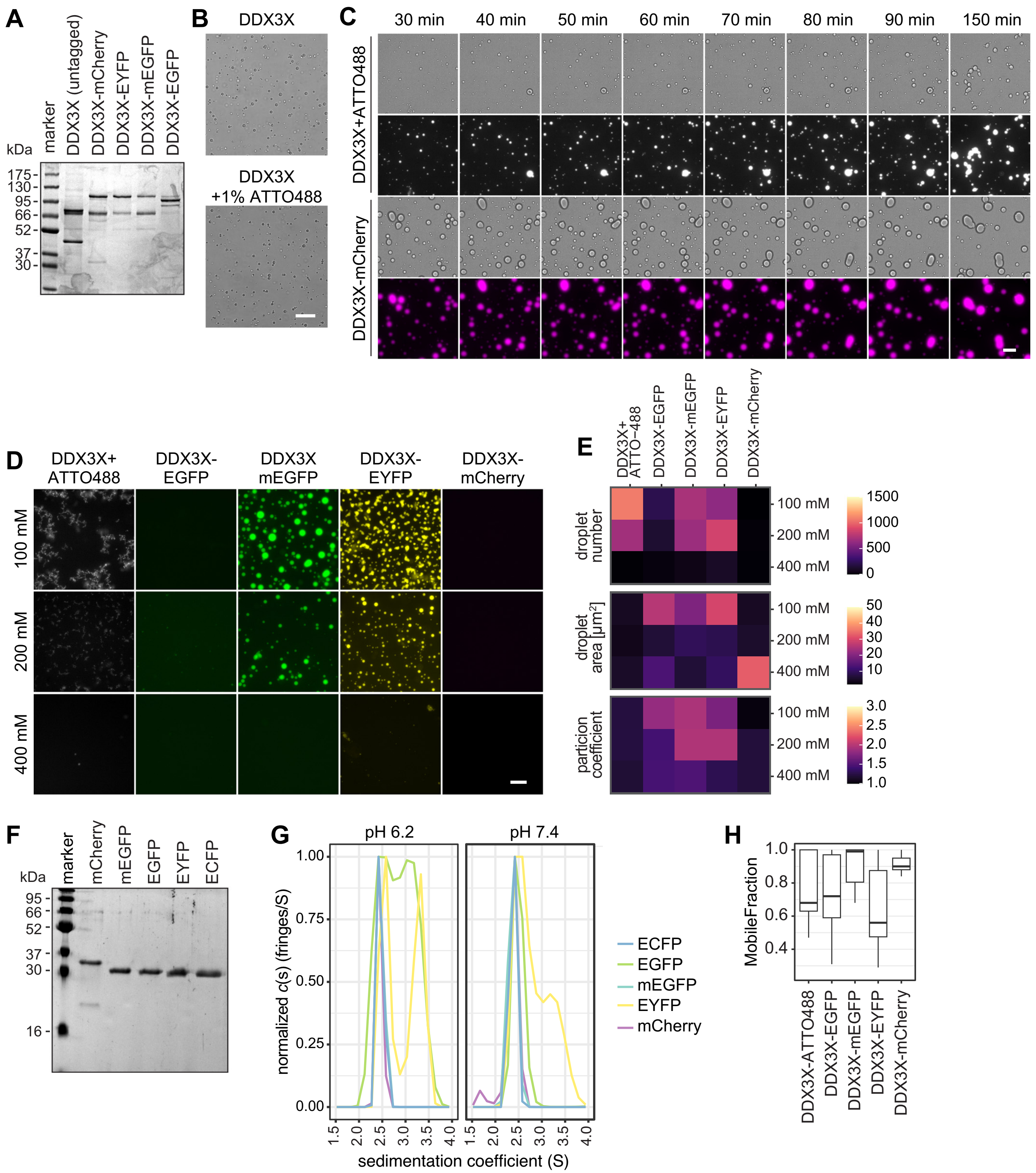
A
marker
DDX3X (untagged)
DDX3X-mCherry
DDX3X-EYFP
DDX3X-mEGFP
DDX3X-EGFP
kDa
175
130
95
66
52
37
30
B
DDX3X
DDX3X +1% ATTO488
C
30 min
40 min
50 min
60 min
70 min
80 min
90 min
150 min
DDX3X+ATTO488
DDX3X-mCherry
D
DDX3X+ ATTO488
DDX3X- EGFP
DDX3X mEGFP
DDX3X- EYFP
DDX3X- mCherry
100 mM
200 mM
400 mM
E
DDX3X+ ATTO-488
DDX3X-EGFP
DDX3X-mEGFP
DDX3X-EYFP
DDX3X-mCherry
droplet number
droplet area [µm²]
particion coefficient
100 mM
200 mM
400 mM
1500
1000
500
0
50
40
30
20
10
3.0
2.5
2.0
1.5
1.0
F
kDa
marker
mCherry
mEGFP
EGFP
EYFP
ECFP
95
66
52
37
30
16
G
pH 6.2
pH 7.4
normalized c(s) (fringes/S)
1.00
0.75
0.50
0.25
0.00
1.5
2.0
2.5
3.0
3.5
4.0
sedimentation coefficient (S)
ECFP
EGFP
mEGFP
EYFP
mCherry
H
MobileFraction
1.0
0.8
0.6
0.4
DDX3X-ATTO488
DDX3X-EGFP
DDX3X-mEGFP
DDX3X-EYFP
DDX3X-mCherry

Figure EV2. Characterization of DDX3X condensates and biophysical properties of fluorescent protein tags.

(**A**) Coomassie stained SDS-PAGE with 0.5 µg of the respective DDX3X protein per lane. (**B**) In vitro condensation assay with 5 µM untagged DDX3X or 5 µM untagged DDX3X + 1% ATTO-DDX3X spike-in in 25 mM sodium phosphate buffer at pH 6.2, 150 mM NaCl, 2 mM $MgCl_2$, 0.5 mg/mL BSA, 0.05 mg/mL poly(U), 0.5 mM ATP, 0.5 mM DTT. Incubated at 25 °C for 30 min before imaging. Scale bar: 20 µm. (**C**) Time series of in vitro condensation assay with 5 µM DDX3X + 1% ATTO488-DDX3X spike-in and DDX3X-mCherry in 25 mM sodium phosphate buffer at the pH 6.2, 150 mM NaCl, 2 mM $MgCl_2$, 0.5 mg/mL BSA, 0.05 mg/mL poly(U), 0.5 mM ATP, 0.05 mM DTT. Incubated at 25 °C for the indicated time before imaging. Scale bar: 20 µm. (**D**) In vitro condensation assay with 5 µM DDX3X (tagged or untagged + 1% ATTO488-DDX3X spike-in) at pH 7.4, 100, 200 or 400 mM NaCl, 2 mM $MgCl_2$, 0.5 mg/mL BSA, 0.05 mg/mL poly(U), 0.5 mM ATP, 0.5 mM DTT. Incubated at 25 °C for 30 min before imaging. Scale bar: 20 µm. (**E**) Quantification of in vitro condensation assay in (**D**): number of condensates per 0.1 mm^2, droplet area and PC, 4 well positions were imaged in three independent replicates. (**F**) Coomassie stained SDS-PAGE with 0.5 µg of the respective FP per lane. (**G**) Analytical ultracentrifugation of 0.5 mg/mL of the respective fluorescent protein in 25 mM phosphate buffer pH 6.2 or 7.4, 150 mM NaCl, 50 mM KCl, 1% glycerol, 1 mM $MgCl_2$ and 0.5 mM β-mercaptoethanol. Values were normalized to the highest value for each construct. (**H**) FRAP of in vitro DDX3X condensates of similar size. 5 µM DDX3X in 25 mM sodium phosphate buffer at pH 6.2, 100 mM NaCl, 2 mM $MgCl_2$, 0.5 mg/mL BSA, 0.05 mg/mL poly(U), 0.5 mM ATP, 0.5 mM DTT. Condensates were matured for 1 h at 25 °C before FRAP. Mobile fraction was analyzed. Box plots show the median (center line), 25th–75th percentiles (bounds of box), and whiskers=1.5x IQR. $N = 3$, $n \geq 23$ condensates.

© The Author(s)

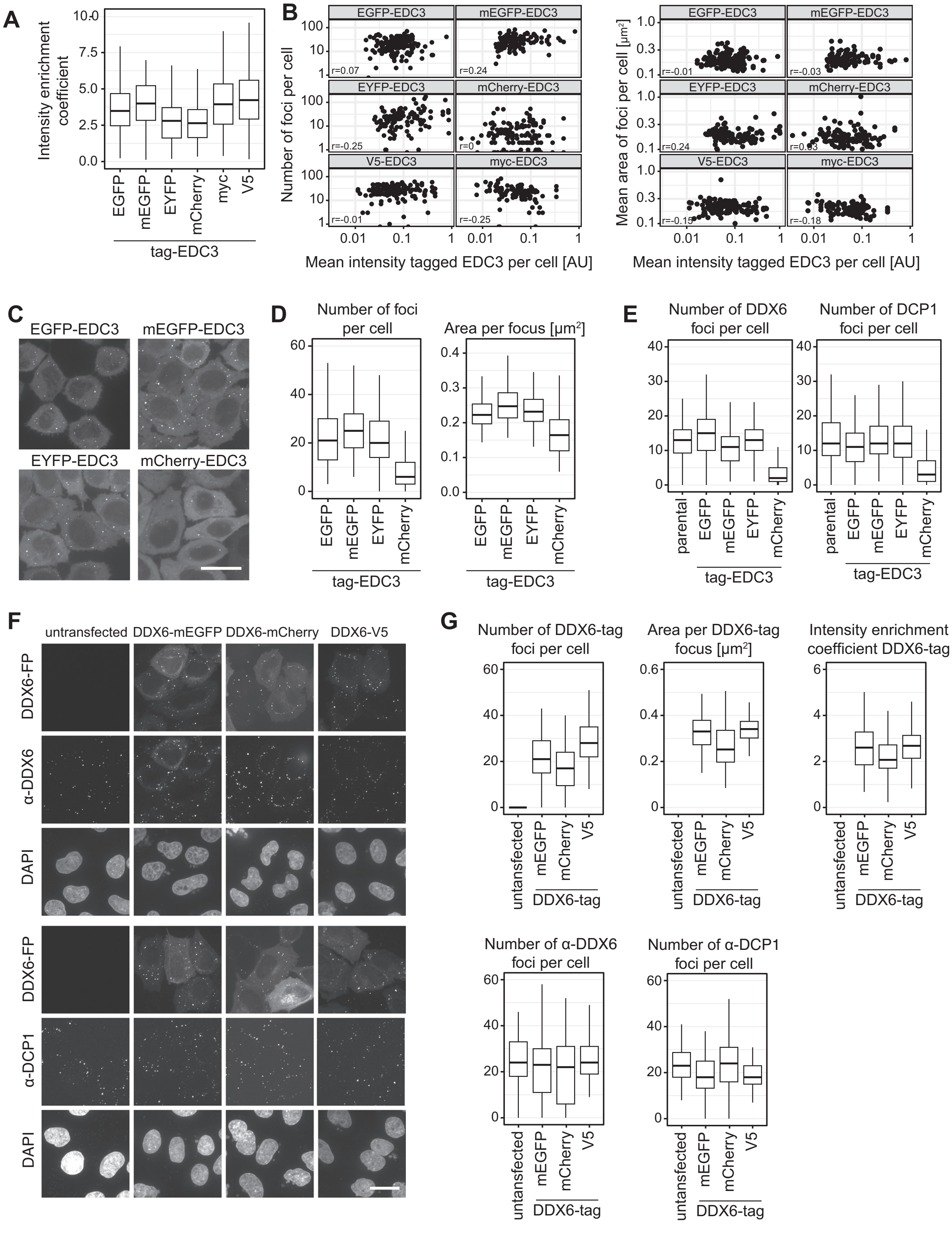
A
Intensity enrichment coefficient
10.0
7.5
5.0
2.5
0.0
EGFP
mEGFP
EYFP
mCherry
myc
V5
tag-EDC3
B
EGFP-EDC3
mEGFP-EDC3
EYFP-EDC3
mCherry-EDC3
V5-EDC3
myc-EDC3
Number of foci per cell
r=0.07
r=0.24
r=-0.25
r=0
r=-0.01
r=-0.25
Mean area of foci per cell [µm²]
r=-0.01
r=-0.03
r=0.24
r=0.03
r=-0.15
r=-0.18
Mean intensity tagged EDC3 per cell [AU]
Mean intensity tagged EDC3 per cell [AU]
C
EGFP-EDC3
mEGFP-EDC3
EYFP-EDC3
mCherry-EDC3
D
Number of foci per cell
Area per focus [µm²]
EGFP
mEGFP
EYFP
mCherry
tag-EDC3
tag-EDC3
E
Number of DDX6 foci per cell
Number of DCP1 foci per cell
parental
EGFP
mEGFP
EYFP
mCherry
tag-EDC3
tag-EDC3
F
untransfected
DDX6-mEGFP
DDX6-mCherry
DDX6-V5
DDX6-FP
α-DDX6
DAPI
DDX6-FP
α-DCP1
DAPI
G
Number of DDX6-tag foci per cell
Area per DDX6-tag focus [µm²]
Intensity enrichment coefficient DDX6-tag
untansfected
mEGFP
mCherry
V5
DDX6-tag
Number of α-DDX6 foci per cell
Number of α-DCP1 foci per cell
untansfected
mEGFP
mCherry
V5
DDX6-tag

◀ **Figure EV3. EDC3 tag effects do not correlate with expression levels, and DDX6 is not influenced by tagging.**

Box plots show the median (center line), 25th–75th percentiles (bounds of box), and whiskers=1.5x IQR. (**A**, **B**) HeLa K cells were transiently transfected with EDC3 plasmids for 24 h, and stressed with 500 µM sodium arsenite for 30 min before fixation. V5-/myc-EDC3 were visualized by immunostaining with the respective antibodies. Quantification of EDC3 foci of cells in Fig. 3A: intensity enrichment coefficient (median intensity per focus/ median intensity of the cell) (**B**), number and area relative to the cellular expression level. Pearson correlation coefficient (r) is indicated. $N = 3$, $n \geq 110$. (**C**, **D**) Stable inducible HeLa cell lines were induced with doxycycline for 24 h to express the respective EDC3-tag constructs. Cells were stressed with 500 µM sodium arsenite for 30 min and imaged live. Scale bar: 20 µm. Quantification of number of EDC3 foci and their area (**D**). $N = 3$, $n \geq 100$ cells. (**E**) Stable inducible HeLa cell lines were induced with doxycycline for 24 h to express the respective EDC3-tag constructs. Cells were stressed with 500 µM sodium arsenite for 30 min before fixation. Cells from the same well were either immunostained for DDX6 or DCP1 with respective antibodies. Scale bar: 20 µm. Quantification of number of DDX6 and DCP1 foci (I). $N = 3$, $n \geq 140$ cells. (**F**, **G**) HeLa K cells were transiently transfected with DDX6 plasmids for 24 h and stressed with 500 µM sodium arsenite for 30 min before fixation. Cells from the same well were either immunostained for DDX6 or DCP1 with respective antibodies. Scale bar: 20 µm. Quantification of number of DDX6 and DCP1 foci (**G**). $N = 3$, $n \geq 85$.

 © The Author(s)

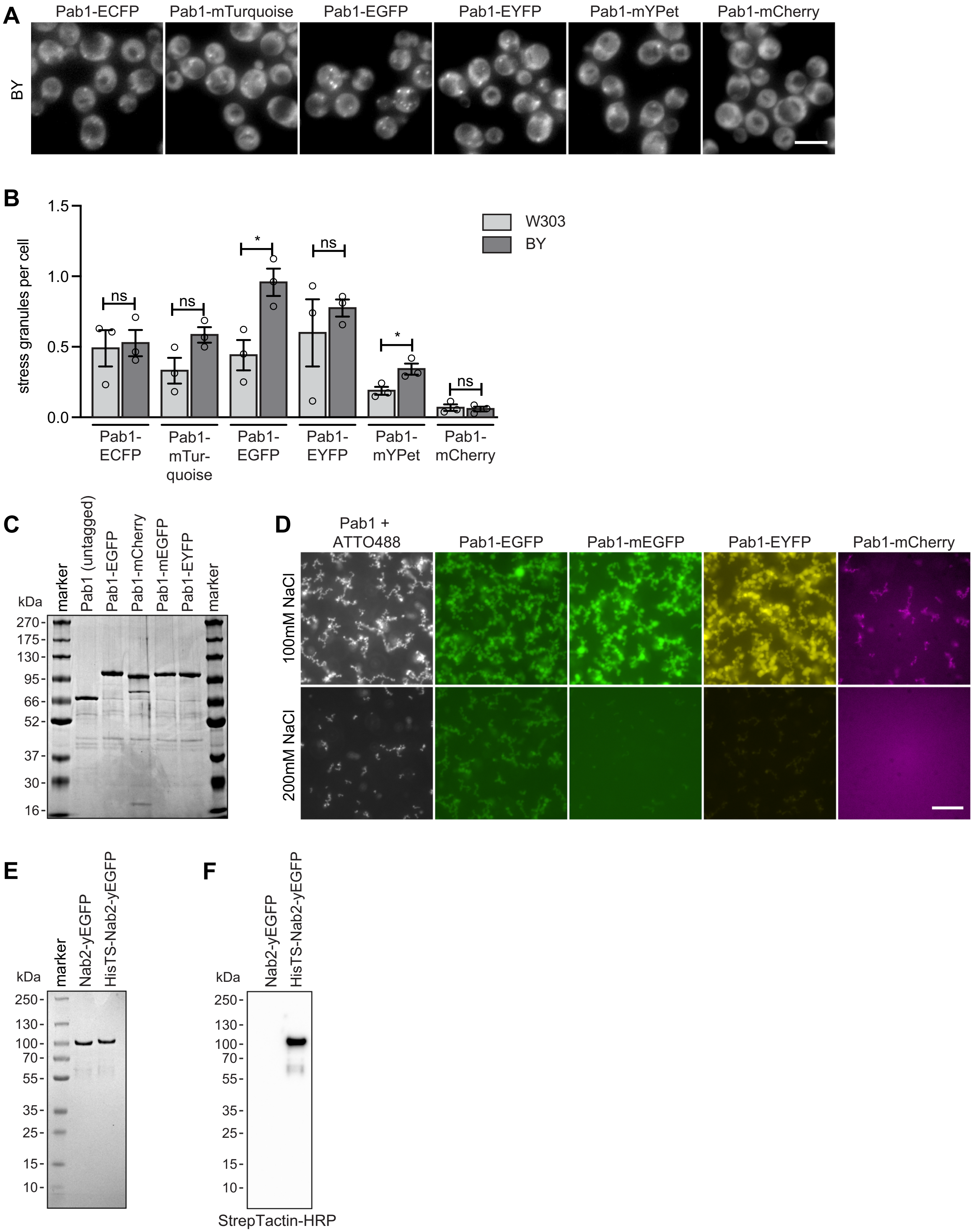
A
Pab1-ECFP
Pab1-mTurquoise
Pab1-EGFP
Pab1-EYFP
Pab1-mYPet
Pab1-mCherry
BY
B
stress granules per cell
1.5
1.0
0.5
0.0
ns
*
W303
BY
Pab1-ECFP
Pab1-mTurquoise
Pab1-EGFP
Pab1-EYFP
Pab1-mYPet
Pab1-mCherry
C
kDa
marker
Pab1 (untagged)
Pab1-EGFP
Pab1-mCherry
Pab1-mEGFP
Pab1-EYFP
marker
270
175
130
95
66
52
37
30
16
D
Pab1 + ATTO488
Pab1-EGFP
Pab1-mEGFP
Pab1-EYFP
Pab1-mCherry
100mM NaCl
200mM NaCl
E
kDa
marker
Nab2-yEGFP
HisTS-Nab2-yEGFP
250
130
100
70
55
35
25
15
10
F
kDa
Nab2-yEGFP
HisTS-Nab2-yEGFP
250
130
100
70
55
35
25
15
10
StrepTactin-HRP

Figure EV4. Pab1 tagging alters condensate formation in yeast and in vitro.

(**A**) *S. cerevisiae* BY strains expressing Pab1 fused with the indicated FP tag were cultivated with 3% glycerol for 45 min prior to live imaging to induce SG formation. p(Pab1-EGFP) = 0.024, p(Pab1-mYPET)=0.034. Representative maximum Z-projections are shown. Scale bar: 5 µm. (**B**) Quantification of SG per cell in (**A**) and Fig. 4A, mean +/− SEM, $N = 3$, $n \geq 1500$ cells per strain. ns: non-significant, $^*P \leq 0.05$ in unpaired *t* test W303 against BY. (**C**) Coomassie stained SDS-PAGE with 0.5 µg of the respective Pab1 protein per lane. (**D**) FP channel images corresponding to DIC images in Fig. 4C. Scale bar: 20 µm. (**E**) Coomassie stained SDS-PAGE with 0.5 µg of the respective Nab2 protein per lane. (**F**) Western Blot with 0.5 µg of the respective Nab2 protein per lane, stained with Strep Tactin conjugated with horse radish peroxidase (HRP).

 © The Author(s)

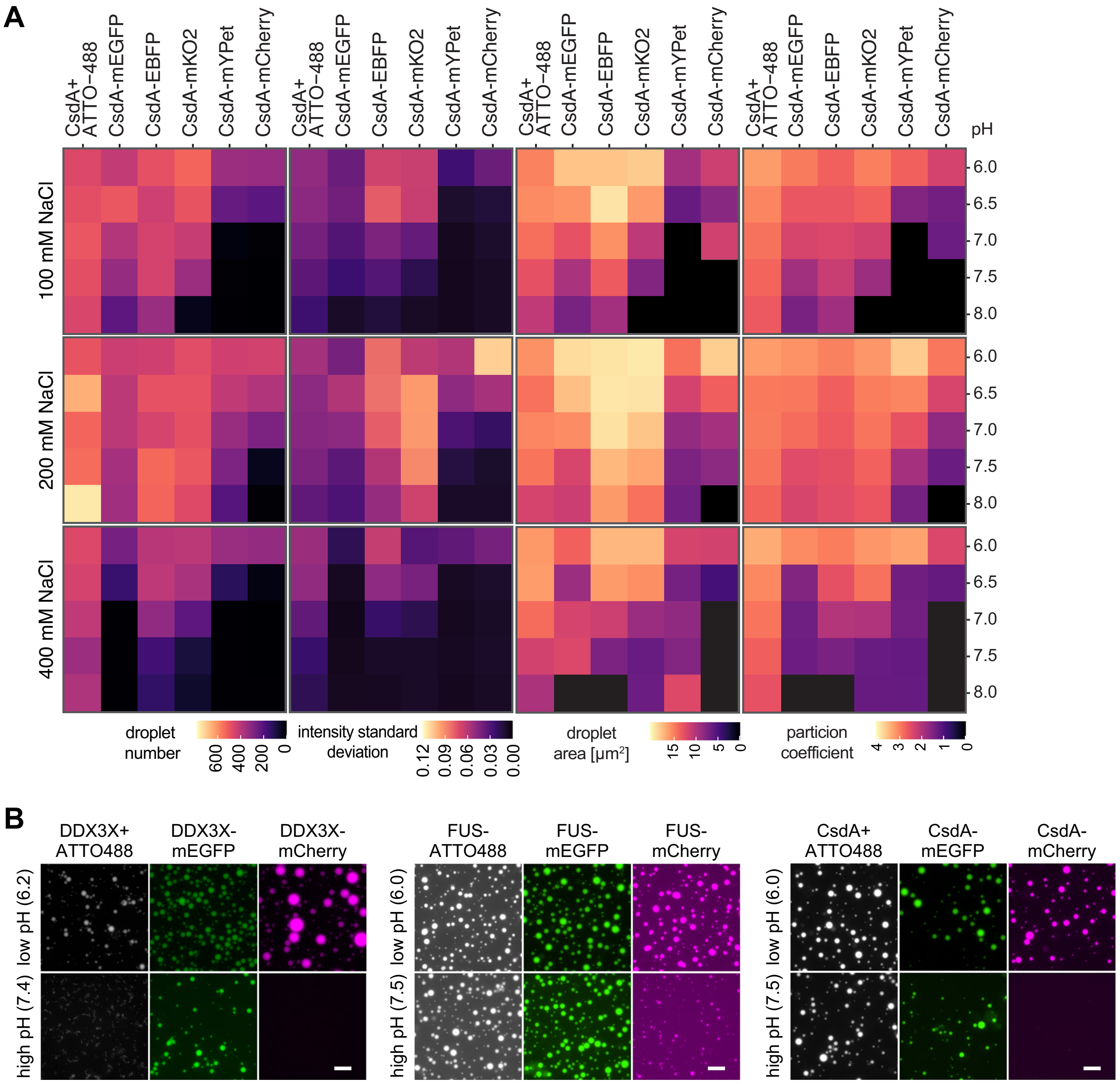

Figure EV5. FP tags and physicochemical parameters modulate CsdA in vitro condensation.

mEGFP and mCherry show distinct pH-dependent effects for DDX3X, FUS, and CsdA. (**A**) Quantification of in vitro condensation assay in (Appendix Fig. S6BA) number of droplets per 0.1 mm^2, droplet area, image intensity standard deviation and PC, $N = 3$. (**B**) Comparative assessment of DDX3X, FUS, and CsdA in vitro condensation when tagged with mEGFP or mCherry at 200 mM NaCl at pH 6.2/7.4 for DDX3X and pH 6.0/7.5 for FUS and CsdA; images are identical to those displayedin Fig. 2C (DDX3X), Fig. 5C (FUS), Appendix Fig. S6B (CsdA). Scale bar: 20 μm.

© The Author(s)

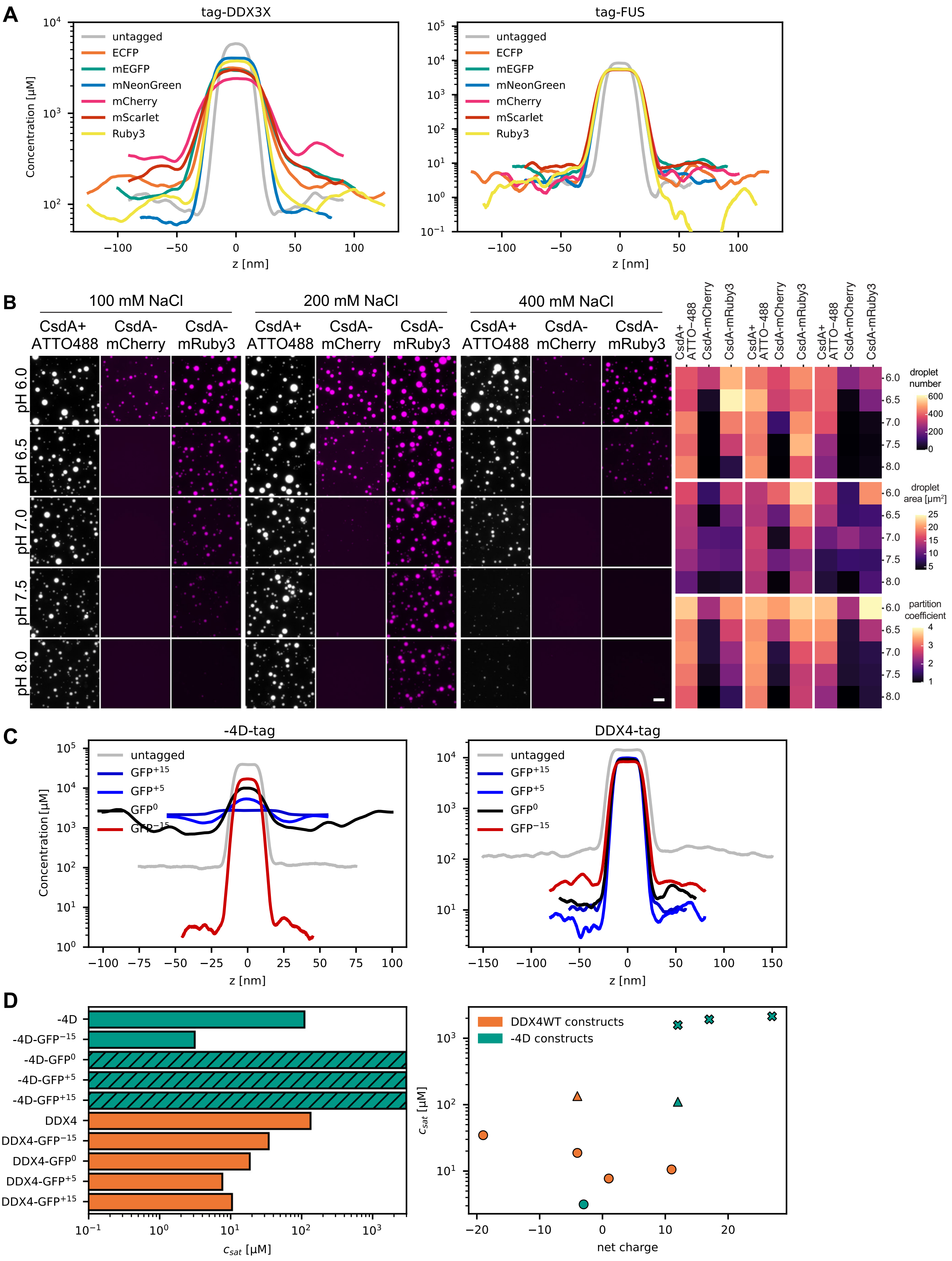
A
tag-DDX3X
tag-FUS
untagged
ECFP
mEGFP
mNeonGreen
mCherry
mScarlet
Ruby3
Concentration [µM]
z [nm]
B
100 mM NaCl
200 mM NaCl
400 mM NaCl
CsdA+ ATTO488
CsdA-mCherry
CsdA-mRuby3
pH 6.0
pH 6.5
pH 7.0
pH 7.5
pH 8.0
droplet number
droplet area [µm²]
partition coefficient
C
-4D-tag
DDX4-tag
untagged
GFP+15
GFP+5
GFP0
GFP−15
Concentration [µM]
z [nm]
D
-4D
-4D-GFP−15
-4D-GFP0
-4D-GFP+5
-4D-GFP+15
DDX4
DDX4-GFP−15
DDX4-GFP0
DDX4-GFP+5
DDX4-GFP+15
csat [µM]
DDX4WT constructs
-4D constructs
net charge

 © The Author(s)

Figure EV6. Phase coexistence simulations indicate that tag position and fluorescent protein charge determine condensation behavior of fusion proteins.

Phase coexistence simulations were performed at 293 K with an ionic strength of 0.15 M using CALVADOS 3. (**A**) Equilibrium density profiles of six FPs N-terminally fused with full-length DDX3X (left) and full-length FUS (right). (**B**) In vitro condensation assay with 2 µM CsdA (tagged or untagged + 1% ATTO488-CsdA spike-in) in 25 mM sodium phosphate buffer at the indicated pHs and NaCl concentrations, 2 mM $MgCl_2$, 0.5 mg/mL BSA, 0.05 mg/mL poly(U). Incubated at 25 °C for 1 h before imaging. Representative images are shown. Scale bar: 20 µm. Quantification: number of droplets per 0.1 mm^2, droplet area and PC, $N = 3$. (**C**) Equilibrium density profiles of the fully intrinsically disordered proteins -4D (left) and truncated DDX4 (right) N-terminally fused with GFP variants with varying net charge. (**D**) Simulated saturation concentrations (c_{sat}, left) and correlation between c_{sat} and net charge of for DDX4 (orange) or -4D (green) constructs with N-terminal tags. Circles represent tagged proteins, triangles untagged proteins. Hatched bars (left) and crosses (right): We did not see a stable condensed phase.
